# Inflammation mediates the relationship between diet quality assessed by healthy eating index-2015 and metabolic syndrome

**DOI:** 10.3389/fendo.2024.1293850

**Published:** 2024-02-05

**Authors:** Li Yuguang, Yu Chang, Hongwei Li, Fangqi Li, Qing Zou, Xiangliang Liu, Xiao Chen, Jiuwei Cui

**Affiliations:** ^1^ Cancer Center, The First Hospital of Jilin University, Changchun, China; ^2^ Department of Anesthesiology, The First Hospital of Jilin University, Changchun, China

**Keywords:** healthy eating index, metabolic syndrome, mediation effect, NHANES, chronic inflammation

## Abstract

**Background:**

Metabolic syndrome is a cluster of metabolic disorders, including obesity, hypertension, hyperglycemia, and abnormal lipid levels. However, researches on the association between overall dietary quality measured by the Healthy Eating Index-2015 (HEI-2015) and the risk of metabolic syndrome is still lacking.

**Methods:**

This study utilized data from four cycles (2011-2018) of the National Health and Nutrition Examination Survey (NHANES) database, including 17,582 participants. Logistic regression analysis was employed to explore the correlation between HEI and the risk of metabolic syndrome. Additionally, mediation analysis was conducted to examine the effects of Systemic Immune-Inflammation Index (SII) and serum uric acid as potential mediators between HEI and metabolic syndrome risk. Weighted quantile sum (WQS) regression evaluated the composite exposure impact of the 13 components of the HEI on metabolic syndrome, as well as the proportion of their weights.

**Results:**

Higher dietary quality measured by HEI-2015 (at the 75th percentile) was negatively correlated with the risk of metabolic syndrome (OR=0.80, 95%CI=0.72-0.89, P=0.003). Higher SII and serum uric acid levels were identified as risk factors for metabolic syndrome (P for trend<0.001). Approximately 37.5% of the effect of HEI on metabolic syndrome occurrence was mediated by SII (Indirect effect=-0.002, 95%CI (-0.003,-0.001), Direct effect=-0.022, 95%CI (-0.0273,-0.015)). Additionally, 25% of the effect of HEI on metabolic syndrome occurrence was mediated by serum uric acid levels (Indirect effect=-0.006, 95%CI (-0.010,-0.012), Direct effect=-0.024, 95%CI (-0.041,-0.009)). WQS regression analysis revealed the highest weighted proportions for seafood and plant proteins (25.20%) and sodium (17.79%), while the weight for whole fruit was the lowest (0.25%).

**Conclusion:**

Better dietary quality measured by HEI-2015 was associated with a lower likelihood of metabolic syndrome. Higher SII and serum uric acid levels were identified as risk factors for metabolic syndrome and potential mediators.

## 1 Introduction

The metabolic syndrome (MetS) refers to a cluster of metabolic abnormalities that increase the risk of cardiovascular disease and type 2 diabetes mellitus (1). The key components of MetS include central obesity, hypertension, dyslipidemia and hyperglycemia (2). With the global epidemic of obesity, the prevalence of MetS has increased substantially over the past few decades. It is estimated that 20-30% of the adult population worldwide has MetS (3). The underlying mechanisms are complex and not yet fully understood, but diet and nutrition have been identified as modifiable factors that may help prevent MetS (4).

The Healthy Eating Index (HEI) developed by the United States Department of Agriculture (USDA) is a measure of diet quality and adherence to the Dietary Guidelines for Americans. The index categorizes food components or nutrients into 13 elements, comprising 9 adequacy components and 4 moderation components, emphasizing a high intake of total vegetables, vegetables and legumes, whole grains, total fruits, whole fruits, total protein foods, plant-based proteins, seafood, and fatty acids and limiting the intake of saturated fatty acids, refined grains, sodium, and added sugars (5). Higher HEI scores indicate closer alignment with key recommendations and better diet quality. Previous studies have shown that higher HEI scores are associated with lower risks of obesity, hypertension, dyslipidemia and diabetes (6–9). However, there is limited research on the association between overall diet quality measured by HEI and risk of MetS, especially in the US population.

Systemic inflammation has been implicated in the pathogenesis of MetS. Chronic, sustained, low-grade inflammation promotes the release of a multitude of inflammatory mediators and cytokines, predisposing the organism to insulin resistance, dyslipidemia, and other metabolic dysregulations (10). Systemic immune-inflammation index (SII), based on peripheral neutrophil, lymphocyte and platelet counts, is a novel composite inflammatory biomarker. Recent evidence suggests that higher SII is associated with increased risks of diabetes, hypertension and metabolic disorders (11–13). Serum uric acid is also linked to systemic inflammation, and hyperuricemia predicts MetS risk (14). Uric acid may mediate the effects of diet on MetS, but few studies have examined its potential mediating role.

In this study, we aimed to investigate the association between HEI and risk of MetS and examine whether SII and serum uric acid levels mediate the association in US adults based on the National Health and Nutrition Examination Survey (NHANES) 2011-2018. The findings will provide novel evidence on the link between diet quality, inflammation and MetS among Americans.

## 2 Methods

### 2.1 Study population

**Figure 1:**
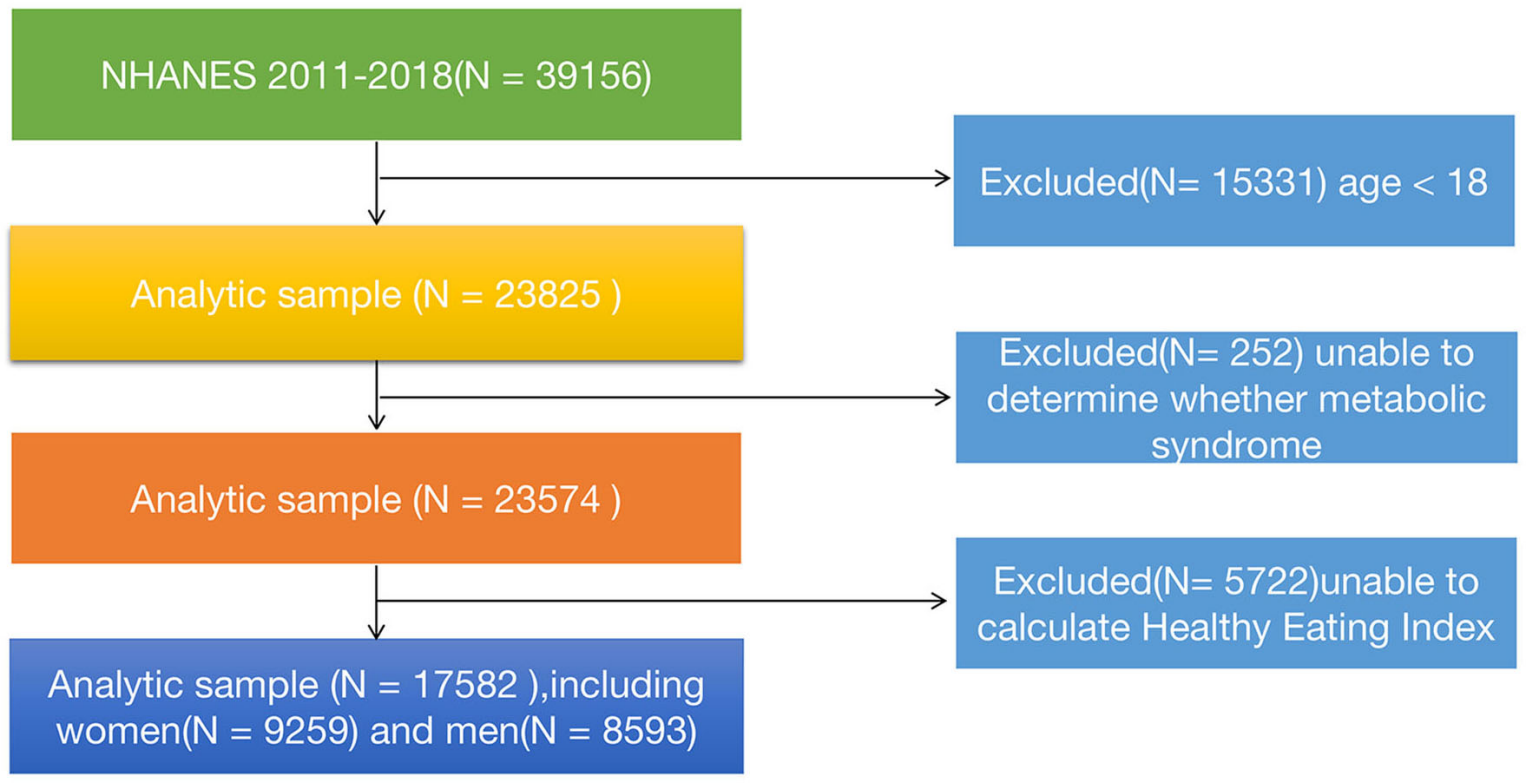
Flowchart of the study design and participants excluded from the study.

In this study, our data was obtained from the publicly available National Health and Nutrition Examination Survey (NHANES) database (https://www.cdc.gov/nchs/nhanes/index.htm). NHANES is a nationally representative cross-sectional survey conducted by the National Institutes of Health and the Centers for Disease Control and Prevention, which aims to comprehensively investigate the health and dietary status of the U.S. population. The database includes modules on demographics, questionnaires, physical examinations, and laboratory tests (including blood and urine samples). For this study, we downloaded four consecutive datasets (2011-2012, 2013-2014, 2015-2016, and 2017-2018) from the website to accurately assess the relationship between a healthy diet index and metabolic syndrome. A total of 39,156 participants’ information was extracted.The following criteria were applied for inclusion and exclusion of participants: Inclusion criteria (1): All participants from 2011 to 2018; (2) Participants who cooperated with the follow-up and provided informed consent. Exclusion criteria: (1) Age< 18 years (n=15,331); (2) Participants with indeterminate MetS status (n=252), including individuals with missing data on blood pressure, total cholesterol, fasting blood glucose, glycated hemoglobin, and body mass index (BMI); (3) Individuals with incomplete or unreasonable dietary data that prevented the calculation of the healthy diet index (n=5,722). After applying these criteria, a total of 17,528 participants were included in this study. The specific details can be shown in **Figure 1**. This NHANES study was approved by the NCHS (National Center for Health Statistics) Ethics Review Board, and informed consent was obtained from all participants.

### 2.2 Data collection

The dietary data for this study was obtained from two 24-hour dietary recall surveys using the Automated Multiple-Pass Method (AMPM) standardized by the USDA (15). In the NHANES survey, trained interviewers conducted face-to-face interviews with participants to collect information on the nutrient composition and intake of various foods and beverages consumed in the past 24 hours. Based on the data from two 24-hour dietary recall surveys, equivalent data grouping transformations and relevant calculations were performed using the USDA Food and Nutrient Database.

The Healthy Eating Index-2015 (HEI-2015) utilizes the population ratio method based on dietary intake data from the entire polulation to assess the reasonableness of the population’s average dietary quality, as well as the adherence to healthy dietary patterns by researchers. This index was jointly developed by the USDA and the Department of Health and Human Services (HHS) in the United States and consists of 13 components including total fruit, whole fruit, total vegetables, greens and beans, dairy, whole grains, protein foods, fats, added sugars, seafood and plant proteins, saturated fats and unsaturated fats, refined grains, and added sugars. These indicators evaluate the diet quality and health of individuals or populations in terms of whole grains, vegetables and fruits, dairy, fats, and sugars, among others. Each component is scored according to specific criteria, and an overall score out of 100 can be calculated. A higher total score indicates greater adherence to the Dietary Guidelines for Americans (DGA) and better overall diet quality (16). The index was calculated and constructed using dietary intake data collected from participants’ two 24-hour dietary recalls, which were combined with USDA Food Patterns equivalent data. We utilized SAS code provided by the National Cancer Institute, and calculated the HEI-2015 score using the HEI scoring algorithm provided by the NHANES database. Detailed components, algorithms, and scoring criteria of HEI-2015 are available in the provided **Supplementary Table 1**.

In this study, participants diagnosed with MetS needed to meet three or more of the following conditions: 1) hypertriglyceridemia: serum triglyceride levels ≥150 mg/dL (1.7 mmol/L), or the use of lipid-lowering medication; 2) low high-density lipoprotein cholesterol levels: HDL<40 mg/dL (1.03 mmol/L) for men, HDL<50 mg/dL (1.29 mmol/L) for women; 3) high blood glucose levels: fasting blood glucose ≥100 mg/dL (5.6 mmol/L), or the use of glucose-lowering medication; 4) hypertension: systolic blood pressure ≥130 mmHg or diastolic blood pressure ≥85 mmHg, or the use of antihypertensive medication; 5) central obesity: waist circumference ≥102 cm for men, ≥88 cm for women (1).

The data for blood samples were measured and recorded by professional researchers utilizing automated hematological analyzers, including platelet count, neutrophil count, lymphocyte count, and uric acid levels. We calculated the Systemic Immune-Inflammation Index (SII), defined as (platelet count × neutrophil count)/lymphocyte count (17). Additionally, we gathered data on gender (female and male), age, race (Mexican American, Non-Hispanic Black, Non-Hispanic White, and other), poverty income ratio (≤1, 1–3, >3), education level (low high school, high school, college or above), smoking status, and alcohol consumption. Current smoker was defined as ‘more than 100 cigarettes in lifetime and currently smoking all or some days’, former smoker as ‘more than 100 cigarettes in lifetime but not currently smoking’, and never smoker as ‘fewer than 100 cigarettes in lifetime’. In terms of alcohol consumption, heavy drinker was defined as ‘weekly alcohol intake exceeding 14 standard drinks for women and 21 standard drinks for men’, moderate drinker as ‘weekly alcohol intake between 8-14 standard drinks for women and between 8-21 standard drinks for men’, and mild drinker as ‘weekly alcohol intake between 1-7 standard drinks’, with one standard drink equivalent to 14 grams of pure alcohol. The HEI-2015 was categorized based on quartiles, Quartile 1 (Q1):<25th percentile, Quartile 2 (Q2): ≥25 to 50th percentile, Quartile 3 (Q3): ≥50 to 75th percentile, and Quartile 4 (Q4): ≥75th percentile. Additionally, both SII and uric acid levels were categorized into quartiles (Q1, Q2, Q3, Q4) using the same approach.

### 2.3 Statistical analysis

Given the complex, multi-stage, stratified sampling design of the NHANES database, we conducted a weighted analysis in accordance with the database’s weighting recommendations. The analysis was weighted. Weighting factors included two-year examination weights (WTMEC2YR) and two-day dietary interview weights (WTDR2D), strata (SDMVSTRA), and primary sampling units (SDMVPSU) were considered to account for the complex survey design. Besides,we employed the data processing method of deleting missing values and accurately documented the number of cases where missing variables were removed. In this study, continuous variables were described using the weighted mean ± standard deviation (Mean ± SD) or the median and interquartile range (M, IQR), statistical differences were assessed using the weighted Student’s t-test or the Kruskal-Wallis rank sum test. Categorical variables were described by the number of samples (weighted percentage), with statistical differences evaluated using Pearson’s Chi-squared test.

For non-normally distributed continuous variables like HEI-2015, SII, and uric acid levels, they were transformed into categorical variables (quartiles) and three multivariable logistic regression models were established to preliminarily assess the relationship between HEI and MetS.These models included: Crude model - not adjusting for any variables; Model 1 - adjusting for gender, age, and race; Model 2 - adjusting for gender, age, race, education level, poverty income ratio, smoking status, and drinking status. Additionally, a second multivariable logistic regression model was used to evaluate the relationship between SII, uric acid levels, and the outcome variable metabolic syndrome. Furthermore, we employed mediation analysis, a method used to determine whether the association between variables can be partially explained by the influence of a third or mediating variable. In this study, the method was conducted to determine whether SII and uric acid levels mediated the relationship between a healthy eating index and MetS. The total effect (TE) represents the direct relationship between HEI-2015 and MetS, unaffected by the mediating factors. The indirect effect (IE) refers to the influence of SII and uric acid levels on MetS through HEI-2015. The direct effect (DE) represents the effect of HEI-2015 on MetS after controlling for SII and uric acid levels. A significant IE indicates the presence of a mediating effect. Besides, We employed sub-group analysis to investigate the relationship between HEI-2015 and MetS across different age groups, genders, races, educational levels, poverty-income ratios and smoking status, and assessed the significance of interaction effects by using p-values of the product terms between HEI-2015 components and stratification factors.

The relationship between mixed exposure of the 13 components constituting HEI and MetS, along with the weight proportion of each component, was analyzed using the WQS regression model.The basic weighted index model is as follows:


$$ɡ\left(\text{µ}\right)=\beta_{o}\;+\beta_{1}\left(\sum_{i=0}^{c}\omega_{i}\varphi_{i}\right)\;+\;z^{\prime }\Phi$$

$$WQS=\sum_{i=1}^{c}{\bar{\omega }}_{i}\varphi_{i}$$

g(µ) denotes any differentiable link function, $\beta_{o}$ represents the intercept, $\beta_{1}$ represents the regression coefficient, c denotes the count of HEI components included in the analysi, $z^{\prime }$ denotes the matrix of covariates, Φ represents the coefficients for these covariates, $\left(\sum_{\text{i}=0}^{\text{c}}{\text{ω}}_{\text{i}}{\text{φ}}_{\text{i}}\right)$ denotes the sum of weighted quantiles for (c) components. $\omega_{i}$ represents the weighted index, where each index ranges from 0 to 1 $\left(0\leq \omega_{i}\leq 1\right)$ , and the total sum of the weighted indices equals 1. $\varphi_{i}$ represents the quartiles of the concentration for each chemical compound, where (i = 0,1,2,3) corresponds to the 1st, 2nd, 3rd, or 4th quartile, respectively (18).

All data analyses in this study were performed using R 4.2.2. All statistical tests were two-sided, and significance was set at P<0.05.

## 3 Results

### 3.1 Baseline characteristics

Participants were grouped based on the presence or absence of MetS, and detailed study characteristics are shown in **Table 1**. A total of 17,528 participants aged over 18 years were included, with 8,269 male participants (47.18%) and 9,259 female participants (52.82%). Among them, approximately 5,901 participants had MetS, while 11,951 participants did not. Compared to the group without metabolic syndrome, individuals with MetS were more likely to be in the age range of 18-65 years, female, non-Hispanic White, with a poverty income ratio (PIR) less than 3, college-educated or higher, non-smokers, drinkers, lower HEI and had higher SII and uric acid levels (p<0.050). For more specific details, please refer to **Table 1**.

**Table 1:** Weighted characteristics of the study population by Metabolic syndrome.

Variable	Level	No Metabolic syndrome	Metabolic syndrome	*P*-value
N		11951	5901	
Sex (%)				0.08
	Female	50.01(0.68)	52.38(1.07)	
	Male	49.99(0.68)	47.62(1.07)	
Age				<0.0001
	[18, 65)	87.17(0.64)	74.66(0.91)	
	≥65	12.83(0.64)	25.34(0.91)	
Race/ethnicity (%)				<0.0001
	Mexican American	8.21(0.85)	9.40(1.10)	
	Non-Hispanic Black	11.33(1.00)	9.18(0.93)	
	Non-Hispanic White	65.71(1.77)	68.79(1.85)	
	Other Hispanic	5.65(0.59)	5.48(0.69)	
	Other Race - Including Multi-Racial	9.10(0.59)	7.14(0.71)	
Poverty (%)				0.003
	<1	14.83(0.90)	13.95(0.87)	
	[1, 3)	34.11(1.18)	38.39(1.15	
	≥3	51.06(1.54)	47.66(1.60)	
Education (%)				<0.0001
	low high school	11.46(0.76)	14.40(0.97)	
	High school	21.25(0.86)	26.46(1.30)	
	College or above	67.29(1.32)	59.14(1.52)	
Smokers(%)				<0.0001
	former smoker	21.21(0.81)	30.29(1.07)	
	Never smoker	60.69(0.97)	52.54(1.08)	
	Current smoker	18.09(0.80)	17.18(0.85)	
Alcohol drinkers(%)				<0.0001
	Former drinker	8.89(0.43)	15.72(0.77)	
	Heavy drinker	22.25(0.82)	17.79(0.97)	
	Mild drinker	37.72(1.08)	38.96(1.54)	
	Moderate drinker	19.97(0.68)	15.41(0.88)	
	Never drinker	11.17(0.83)	12.12(0.93)	
HEI(mean (SD))		51.94(0.35)	51.01(0.34)	0.01
SII (mean (SD))		497.62 (332.28)	539.48 (325.84)	<0.0001
Uric acid(mean (SD))		506.25(5.48)	553.29(5.90)	<0.0001

Mean ± SE for continuous variables: P-value was calculated by the weighted T test. % (SE) for categorical variables: P-value was calculated by the weighted chi-square test.

### 3.2 Association between HEI and metabolic syndrome

Three multivariable logistic regression models were established to further investigate the impact of HEI on MetS. Compared to the Q1 group, the Q4 group showed a negative correlation with the occurrence of metabolic syndrome. In Model 1, compared to the Q1 quartile, the risk of developing MetS in the Q4 quartile was reduced by 22% (OR 0.78, 95% CI 0.66-0.92, p=0.003). In Model 2, compared to the Q1 quartile, the risk of developing MetS in the Q4 quartile was reduced by 23% (OR 0.77, 95% CI 0.66-0.91, p=0.003). Further details can be seen in **Table 2**.

**Table 2:** The associations between HEI and metabolic syndrome.

	Crude model^a^		Model 1^b^		Model 1^c^	
	OR(95% CI)	*P*-value	OR(95% CI)	*P*-value	OR(95% CI)	*P*-value
HEI						
Q1	Reference		Reference		Reference	
Q2	1.02(0.86,1.21)	0.80	0.97(0.82,1.16)	0.73	0.98(0.82,1.16)	0.80
Q3	1.00(0.85,1.17))	0.95	0.91(0.77,1.08)	0.27	0.92(0.77,1.10)	0.35
Q4	0.87(0.74,1.02)	0.08	0.78(0.66,0.92)	0.003	0.77(0.66,0.91)	0.003

Q, quartiles; Q1 represents the unhealthiest diet quality, Q4 represents the healthiest diet quality. OR, odds ratio; CI, confidence intervals. Crude model^a^: no covariates were adjusted. Model 1^b^: sex,age and race/ethnicity were adjusted. Model 2^c^: sex,age, race/ethnicity, education attainment, poverty income ratio, smoking status, and alcohol drinking status were adjusted.

### 3.3 The relationship between SII, uric acid levels, and metabolic syndrome

In order to further investigate the relationship between SII, uric acid levels, and MetS, and the role of covariates in the model, we categorized SII and uric acid levels into quartiles. We adjusted for gender, age, race, education level, poverty-income ratio, smoking status, and alcohol consumption in logistic regression models. Compared to the Q1 quartile for SII, the risk of developing MetS in the Q2, Q3, and Q4 quartiles increased by 21% (OR 1.21, 95% CI 1.06-1.38, p=0.010), 35% (OR 1.35, 95% CI 1.16-1.57, p<0.001), and 66% (OR 1.66, 95% CI 1.44-1.91, p<0.001), respectively. Compared to the Q1 quartile for uric acid levels, the risk of developing MetS in the Q2, Q3, and Q4 quartiles increased by 92% (OR 1.92, 95% CI 1.64-2.25, p<0.001), 184% (OR 2.84, 95% CI 2.37-3.40, p<0.001), and 404% (OR 5.04, 95% CI 4.25-5.98, p<0.001), respectively. Therefore, both SII and uric acid levels can be considered important risk factors for the occurrence of MetS. More details can be observed by referring to **Table 3**.

**Table 3:** The correlation between SII, uric acid levels, and metabolic syndrome.

	OR	95% CI	*P*-value
SII			
Q1	Reference		
Q2	1.17	(1.06,1.30)	0.003
Q3	1.25	(1.13,1.39)	<0.001
Q4	1.44	(1.30.1.60)	<0.001
Uric acid			
Q1	Reference		
Q2	1.76	(1.58,197)	<0.001
Q3	2.62	(2.23,2.93)	<0.001
Q4	4.46	(3.96,5.02)	<0.001

Adjusted for sex,age, race/ethnicity, education attainment, poverty income ratio, smoking status, and alcohol drinking statuss. Q, quartiles; Q1 represents the unhealthiest diet quality, Q4 represents the healthiest diet quality. OR, odds ratio; CI, confidence intervals.

### 3.4 Mediation analysis

**Figure 2:**
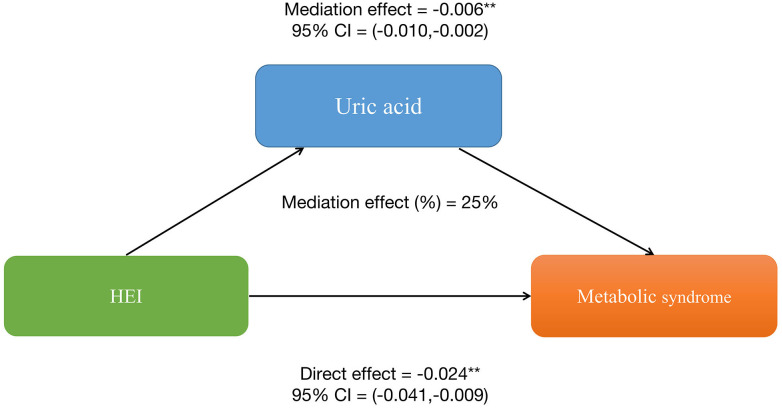
Serum uric acid levels partially mediates the relationship between healthy eating index-2015 and metabolic syndrome. *P<0.05, **P<0.01.

To explore whether SII and uric acid mediate the relationship between HEI and MetS, we conducted a mediation analysis. In this mediation model, HEI was treated as the independent variable, MetS as the dependent variable, and SII and uric acid levels as the mediating variables. The results are shown in **Figure 2**: HEI has a significant indirect effect on the occurrence of MetS through serum uric acid levels, with an indirect effect of -0.006 (95%CI: -0.010, -0.012). This suggests that serum uric acid levels partially mediate the relationship between HEI and MetS. After controlling for serum uric acid levels, HEI still exerts a significant inhibitory effect on the occurrence of MetS, with a direct effect of -0.024 (95%CI: -0.041, -0.009). This indicates that there are both direct and indirect effects of HEI on the occurrence of MetS. Approximately 25% of the impact of HEI on the occurrence of MetS is mediated by serum uric acid levels.

**Figure 3:**
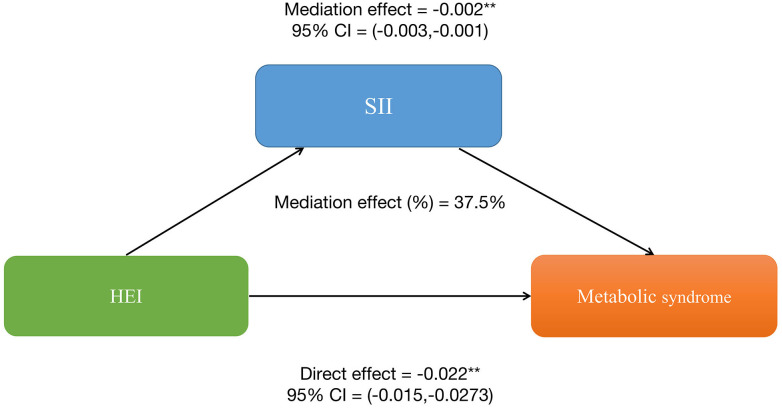
Systemic Immune-Inflammation Index (SII) partially mediates the relationship between healthy eating index-2015 and metabolic syndrome. *P<0.05, **P<0.01.

Similarly, HEI also has a significant indirect effect on the occurrence of MetS through SII, with an indirect effect of -0.002 (95%CI: -0.003, -0.001), suggesting a partial mediating effect of SII. After controlling for SII, HEI still shows a significant inhibitory effect on the occurrence of MetS, with a direct effect of -0.022 (95%CI: -0.0273, -0.015). This further indicates the presence of both direct and indirect effects of HEI on the occurrence of MetS, with approximately 37.5% of the impact of HEI on MetS occurrence being mediated by SII. More details can be shown in **Figure 3**. Therefore, both SII and serum uric acid levels can be considered important mediating factors in the relationship between HEI and the occurrence of MetS.

### 3.5 Subgroup analysis and interaction

**Figure 4:**
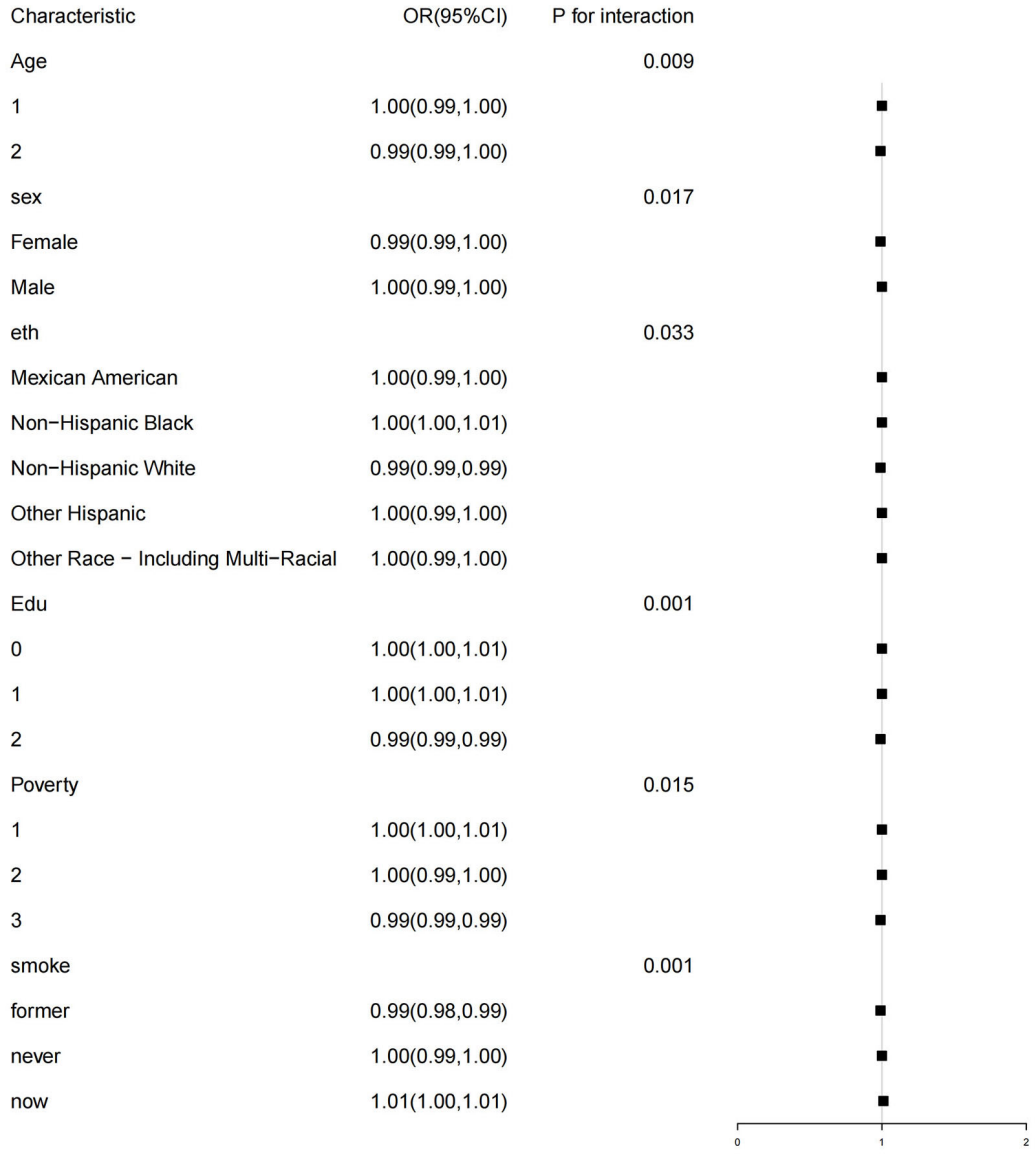
WQS model regression index weights for the metabolic syndrome (MetS), adjusted for sex,age, race/ethnicity, education attainment, poverty income ratio, smoking status, and alcohol drinking status.

The subgroup analysis revealed a significant interaction between HEI-2015 and age, gender, education level, PIR, and smoking status (p<0.05). The negative correlation between HEI-2015 and MetS was observed in older participants (aged > 65 years, OR 0.99, 95%CI (0.90,1.00)), female participants (OR 0.99, 95%CI (0.99,1.00)), non-Hispanic White participants (OR 0.99, 95%CI (0.99,0.99)), participants with a university or higher education level (OR 0.99, 95%CI (0.99,0.99)), and participants with a PIR≥3 (OR 0.99, 95%CI (0.99,0.99)). For more specific details, please refer to **Figure 4**.

### 3.6 Weighted quantile sum regression

**Figure 5:**
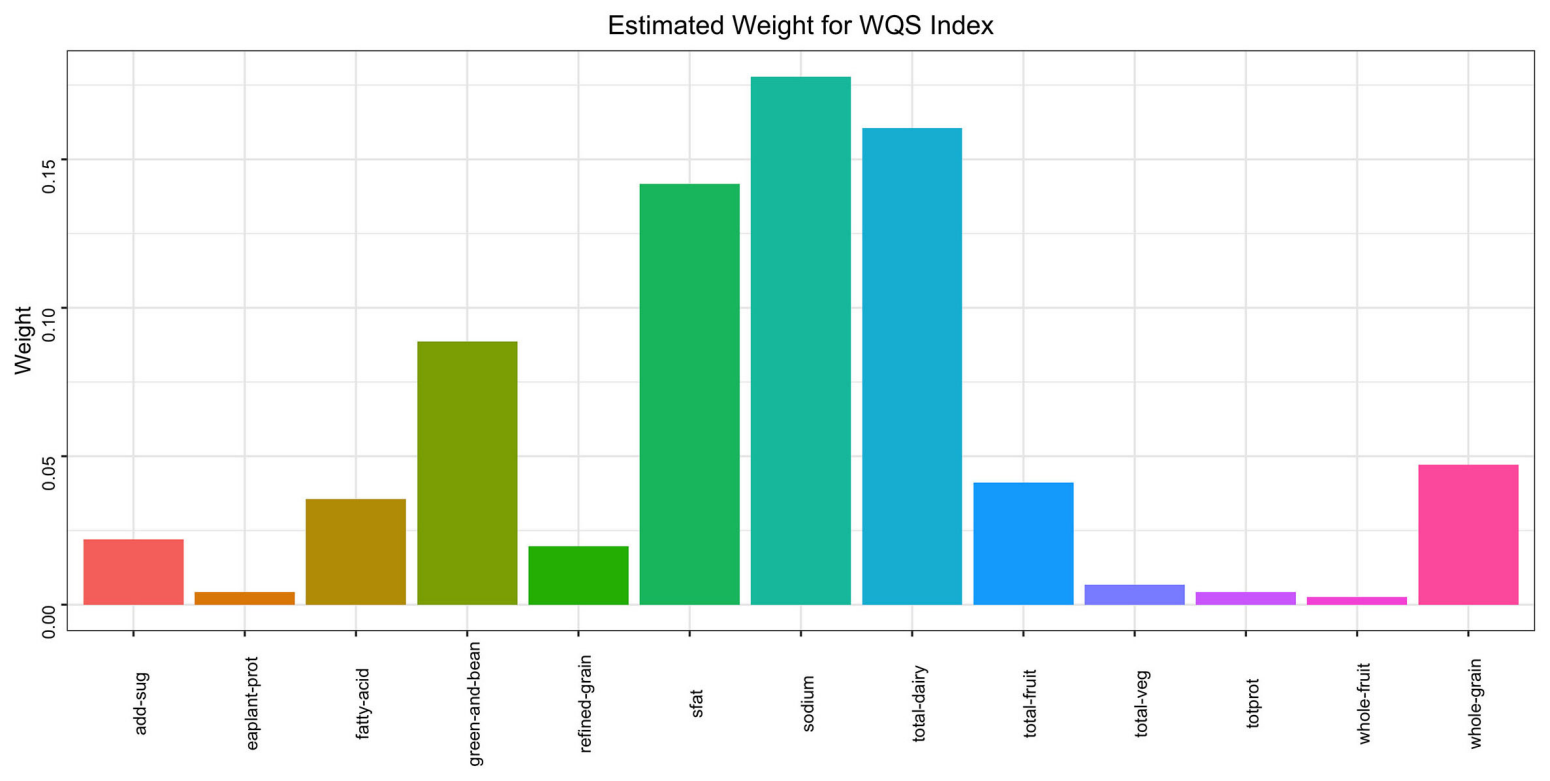
Subgroup analysis of the association of the HEI-2015 and the presence of metabolic syndrome (MetS). Each stratifcation was adjusted for age, sex, race/ethnicity, education attainment, poverty income ratio, smoking status. Age (1 aged 18-65 years; 2 ≥65years), Edu Education level (0 low high school; 1 high school; 2 College or above), Poverty (1<1; 2 [1, 3); 3 ≥3).

The WQS regression model was employed to construct a weighted index for investigating the cumulative impact and proportional weights of the 13 components of the HEI on the risk of MetS. The findings illustrated a statistically significant inverse association between the overall HEI score and the prevalence of MetS (OR = -0.47, P< 0.001). Among the 13 components of the HEI, seafood and plant proteins (25.20%) along with sodium (17.79%) had the highest weights, whereas whole fruit had the lowest (0.25%). More detailed information can be obtained in **Figure 5**.

## 4 Discussion

In this large, nationally representative sample of US adults, we found that better diet quality measured by HEI-2015 was associated with lower odds of having MetS. The association remained significant after adjusting for demographics, socioeconomics, smoking and alcohol use. In addition, higher SII and serum uric acid levels were identified as risk factors for MetS. Mediation analysis further revealed that SII and uric acid partially mediated the association between HEI and MetS. The WQS regression model assessed the cumulative effects and weights of the 13 HEI components on MetS, with substantial consumption of seafood and plant proteins and moderate intake of sodium being most significantly associated with a reduced risk of MetS.

Our study provides new evidence that better adherence to the Dietary Guidelines for Americans and higher diet quality are associated with reduced Mets risk. The HEI-2015 assesses conformity to key dietary recommendations on fruits, vegetables, whole grains, dairy, protein, fatty acids, refined grains, sodium and empty calories (5). Meeting these targets may help prevent development of individual MetS components including central obesity, hypertension, dyslipidemia and hyperglycemia. In alignment with our findings, previous studies have linked higher HEI scores to lower prevalence of obesity, diabetes, hypertension and dyslipidemia (6–9). Two separate studies, one focusing on Korean adults and the other on Iranian adults, have also reported the associations between HEI scores and MetS risk (19, 20). Our research further confirmed the beneficial effects of diet quality in MetS among Americans. Interventions to improve diet quality should be a public health priority.

Chronic low-grade inflammation is involved in MetS pathogenesis (4). SII and uric acid are two emerging inflammatory markers associated with obesity, insulin resistance, diabetes and cardiovascular disease (11–14). Our study newly identified higher SII and uric acid as independent predictors of MetS after adjusting for confounders. Inflammation may be a key mechanism linking diet quality to MetS. Mediation analysis showed that the associations between HEI and MetS were partially mediated by SII and uric acid, suggesting both direct and indirect effects. A high-quality diet may help decrease systemic inflammation, thereby lowering MetS risk. The mediating roles of novel inflammatory markers warrant further investigation in diet and MetS research.

Compared to other diet quality indices, the HEI-2015 is unique as it quantifies conformance to federal dietary guidelines. However, associations between diet quality and inflammation/MetS likely reflect commonalities across indices like higher intakes of plant foods and unsaturated fats versus processed foods and saturated fats. For instance, the Mediterranean diet characterized by high intakes of fruits, vegetables, legumes, cereals, fish, and monounsaturated fatty acids has been linked to lower systemic inflammation and MetS prevalence (21, 22). The DASH diet targeting high intake of fruits, vegetables, low-fat dairy, whole grains, chicken, fish, nuts and low intake of red meats, sweets, and sugar-sweetened beverages also reduces CRP and other inflammatory markers (23). Overall diet quality appears more influential than individual components. Further research can continue examining how different diet quality indices relate to inflammation, MetS risks, and cardiometabolic health. Our study utilized the HEI to evaluate diet quality in relation to MetS risk among Americans, providing dietary strategies for MetS prevention.

Our research found that SII and uric acid partially mediated the relationship between HEI and MetS. The potential beneficial effects of diet quality on mitigating inflammation and MetS risk likely accumulate over time. Long-term adherence to high-quality diets emphases fruits, vegetables, fiber, plant proteins, and unsaturated fats while limiting red meats, saturated fats, processed foods, and sugars. These healthy dietary patterns characterized by high HEI scores have been associated with lower systemic inflammation marked by CRP, interleukin-6, E-selectin and other biomarkers in both cross-sectional and prospective studies (24–26). The compounding anti-inflammatory effects could alleviate insulin resistance, endothelial dysfunction, and dyslipidemia central to MetS pathogenesis (27). For example, the phytochemicals and antioxidants in plant foods may improve insulin signaling and glucose metabolism through regulating oxidative stress and inflammatory pathways (28). Omega-3 fatty acids can inhibit nuclear factor kappa B activation, suppressing production of inflammatory cytokines like tumor necrosis factor alpha and interleukin-6 (29). The high fiber content in whole grains, fruits and vegetables may also contribute by producing short-chain fatty acids upon fermentation, which hold immunomodulatory activities (30), lower levels of purines, thus reducing the production of uric acid. These plant-based foods are abundant in vitamins C and E, and marine foods are rich in polyphenols and flavonoids. The rich content of these bioactive compounds exerts potent anti-inflammatory and antioxidant effects, mitigating systemic immune-inflammatory responses, thereby lowering the SII (31, 32). Chronic inflammation can interfere with the normal function of insulin, leading to insulin resistance; the release of inflammatory cells and mediators contributes to dysregulated lipid metabolism, increasing the risk of MetS (11). Elevated uric acid levels may promote oxidative stress and cellular apoptosis, heightening the incidence of hypertension, gout, and cardiovascular diseases (33).In addition, higher HEI signifies more balanced nutrition intake, which can improve insulin sensitivity and reduce inflammatory cytokine production (21, 34). Future research is warranted to investigate the anti-inflammatory effects of high-quality diets and their impacts on MetS.

Our study boasts several innovative advantages, firstly, it is based on a large national NHANES sample, ensuring ample sample size and validity of the research outcomes. We constructed several weighted multivariate logistic regression models, adjusting for multiple variables to robustly assess the impact of HEI on the risk of incident MetS. Moreover, we utilized mediation analysis to further elucidate the potential mediating roles of SII and uric acid in the association between HEI and MetS. We employed the weighted quantile sum regression approach to test the cumulative effects of the 13 components of HEI on MetS and quantified the significant contributions of each component, substantially advancing the reliability and robustness of dietary characteristics associated with MetS.

This study has important public health implications regarding the role of diet quality in metabolic health. Our findings that better adherence to federal dietary guidelines assessed by HEI-2015 is associated with lower likelihood of MetS in Americans further highlights the value of the HEI as a diet quality monitoring tool. The index could help shape dietary recommendations and policies aimed at curbing the growing prevalence of obesity, diabetes, hypertension and related metabolic disorders. Specifically, the HEI-2015 components with the greatest weights in relation to MetS risk in our analysis—including seafood/plant proteins, sodium and empty calories—can inform targeted efforts to improve these aspects of diet quality at the population level. Future revisions to federal dietary guidelines and educational campaigns can also emphasize overall diet patterns aligned with HEI-2015 targets rather than individual nutrients or foods.

However, our study has some limitations. The cross-sectional analysis prevents causal determination of the relationship between diet quality and MetS. While the NHANES dataset is nationally representative, the self-reported dietary data may be subject to recall biases. We identified certain novel inflammatory biomarkers as mediators but did not measure an exhaustive profile. Future longitudinal cohorts could track participants’ diets using validated methods alongside periodic assessment of clinical parameters, MetS incidence and detailed inflammatory markers to elucidate long-term relationships. Examining how diet quality interacts with genetics, microbiome and other factors influencing MetS risks would also advance scientific understanding. Overall, our findings highlight diet quality as a modifiable factor and the utility of HEI-2015 to assess adherence to healthy dietary patterns for lowering metabolic disease burden.

## 5 Conclusion

In conclusion, we found that better diet quality assessed by HEI-2015 was associated with lower likelihood of having MetS in a nationally representative sample of US adults. Higher SII and serum uric acid levels were identified as risk factors for MetS and partial mediators. Our results highlight the importance of a high-quality diet and controlling inflammation in MetS prevention. The HEI-2015 index may be a useful tool to monitor diet quality at the population level. Our findings need to be confirmed by longitudinal studies elucidating the interrelationships between diet, inflammation and metabolic disorders. Nutrition strategies to improve diet quality, reduce inflammation and alleviate the growing burden of MetS warrant further investigation.

## Data availability statement

The raw data supporting the conclusions of this article will be made available by the authors, without undue reservation.

## Ethics statement

The studies involving humans were approved by the National Center for Health Statistics Research Ethics Review Board, duly approved by the ethical review committee (protocol #2011-17, #2018-01). The patients/participants provided their written informed consent to participate in this study.

## Author contributions

LY: Data curation, Formal analysis, Visualization, Writing – original draft. YC: Data curation, Formal analysis, Visualization, Writing – original draft. HL: Data curation, Formal analysis, Writing – original draft. FL: Methodology, Project administration, Writing – original draft. QZ: Methodology, Project administration, Writing – original draft. XL: Conceptualization, Methodology, Supervision, Writing – original draft, Writing – review & editing. XC: Conceptualization, Supervision, Writing – review & editing. JC: Conceptualization, Methodology, Supervision, Writing – review & editing.
